# Effect of Potassium Ions on the Formation of Mixed-Valence Manganese Oxide/Graphene Nanocomposites

**DOI:** 10.3390/ma12081245

**Published:** 2019-04-16

**Authors:** Wooree Jang, Dae-Young Jeon, Youn-Sik Lee, Hye Young Koo

**Affiliations:** 1 Functional Composite Materials Research Center, Korea Institute of Science and Technology (KIST) Jeonbuk Institute of Advanced Composite Materials, 92 Chudong-ro, Bongdong-eup, Wanju-gun, Jeollabuk-do 55324, Korea; 215015@kist.re.kr (W.J.); dyjeon@kist.re.kr (D.-Y.J.); 2 School of Chemical Engineering, Chonbuk National University, Jeon-ju, Jeollabuk-do 54896, Korea

**Keywords:** manganese oxide, reduced graphene oxide, nanocomposite, graphene

## Abstract

One-pot synthesis of mixed-valence manganese oxide (MnO_x_)/potassium ion-doped reduced graphene oxide (rGO) composites for efficient electrochemical supercapacitors is introduced. Using manganese nitrate and potassium permanganate as co-precursors for the MnO_x_ and by directly annealing the rGO without tedious purification steps, as described herein, MnO_x_/rGO composites with a high specific capacitance of 1955.6 F g^−1^ at a current density of 1 A g^−1^ are achieved. It is found that the presence of potassium ions helps in the development of mixed-valence MnO_x_ on the surface of the rGO.

## 1. Introduction

Recently, composite electrode materials comprising electrochemical double-layer capacitors (EDLCs) and pseudocapacitors have come to represent a promising avenue for providing greater energy density for supercapacitors. The use of a pseudocapacitive material enables fast electron transfer at the surface of the electrode, leading to a higher energy density than can be achieved by using EDLCs alone. As a promising candidate for a composite electrode material, nanocomposites composed of manganese oxide (MnO_x_) and graphene have been widely investigated [1,2,3,4,5,6,7,8]. The properties of graphene, such as high electrical conductivity, large specific surface area, transparency, and flexibility, are beneficial to use as a supercapacitor electrode material [9,10,11,12,13,14,15,16,17]. Manganese oxide is attractive as a pseudocapacitor because of its low cost, natural abundance, environmental compatibility, and high theoretical specific capacitance [18]. The shortcoming of manganese oxide as a supercapacitor electrode is its lack of electrical conductivity, which can be compensated for by the use of graphene composites. In addition, the high surface area and high electrical conductivity of graphene can improve the electrochemical performance by double layer formation, and this is also helped by a uniform distribution of the manganese oxides on the basal plane of the graphene.

The electrochemical performance of the manganese oxides are directly influenced by their crystal structures and oxidation states. Several studies have reported on how the electrochemical performance depends on the oxidation states of manganese oxides [19,20]. In particular, it is reported that the presence of mixed-valence manganese oxides can result in superior performance of capacitive behavior, due to the coexistence of aliovalent manganese cations that drive the defect-accelerated kinetics of the surface reactions [21]. 

In this study, we report on a one-pot synthesis of a mixed-valence manganese oxide (MnO_x_)/potassium (K^+^) ion-doped, reduced graphene oxide (rGO) composite materials for an efficient supercapacitor electrode. By using manganese nitrates and potassium permanganate as co-precursors for MnO_x_ and then using direct annealing without any intervening washing steps, mixed-valence MnO_x_/rGO composites with a K^+^-doped rGO structure were successfully achieved. Due to the synergetic effects created by the presence of mixed-valence MnO_x_ and K^+^ ions, the resulting composite structure showed excellent capacitive properties, reaching a maximum specific capacitance of 1955.6 F g^−1^ at a current density of 1 A g^−1^.

## 2. Materials and Methods

### 2.1. Materials

H_2_SO_4_ (98%), H_2_O_2_ (35%), and HCl (5%) were purchased from Dae-Jung (Suwon, Korea). Graphite flakes, KMnO_4_, manganese (II) nitrate tetrahydrate (Mn(NO_3_)_2_·4H_2_O), KOH, activated carbon, N-methyl-2-pyrrolidone (NMP), and polyvinylidene fluoride (PVDF) were purchased from Sigma-Aldrich (Steinheim, Germany). All of these materials were used without any further purification.

### 2.2. Preparation of GO

A modified Hummers’ method was used to prepare the Graphite oxide (GO), as described in previous reports [22]. Briefly, 2 g of graphite flakes were mixed with 46 mL of 98% sulfuric acid in a 250 mL round bottom flask and placed in an ice bath with constant stirring. Then 6 g of potassium permanganate was slowly added to the mixture. After 2 h, the supernatant mixture was transferred to an oil bath and kept at a constant temperature of 35 °C for 6 h. Then 92 mL of de-ionized (DI) water was gradually added to the reaction mixture. The mixture was then stirred for 1 h. The whole reaction mixture was then poured into a 1 L beaker containing 240 mL of water. Then 35% hydrogen peroxide solution was added until the color of the mixture changed to bright yellow. Hydrochloric acid diluted in 5% water was added in order to remove the metal cations. Finally, the resulting solution was washed with DI water, and dialysis was performed until a neutral graphite oxide solution was obtained. 

### 2.3. Preparation of MnO_x_/rGO Nanocomposites

100 mg of GO was uniformly dispersed in 200 mL of DI water using water bath sonication for 1 h. Then 20 mL each of 0.01 M Mn(NO_3_)_2_·4H_2_O and KMnO_4_ solutions were simultaneously added, dropwise, to the GO solution with stirring at a constant speed for 1 h. The supernatant mixture was then dried at room temperature, and finally the remaining moisture was removed by vacuum drying at room temperature. No washing with water was involved in this process. The resulting solid powder-type mixtures were annealed at 500 °C in an Ar atmosphere. For a comparative study, samples annealed at 400 °C and 600 °C were also prepared and compared for their electrochemical performances.

### 2.4. Characterization

A Tecnai G2 F20 (FEI, Hillsboro, OR, USA) was used to take transmission electron microscopy (TEM) images and perform energy dispersive X-ray spectroscopy (EDS) analysis. X-ray diffraction (XRD) patterns were recorded on a Rigaku SmartLab diffractometer (Rigaku, Tokyo, Japan) using Cu Kα radiation. A Jobin Yvon LabRAM HR UV-Visible-NIR spectrometer (Horiba, Kyoto, Japan) was used to obtain Raman spectra. X-ray photoelectron spectroscopy (XPS) information was obtained on a Thermo Fisher Scientific (Waltham, MA, USA) K-alpha using an Al source. Fourier transform infrared (FT-IR) spectra were recorded on a FR/IR-6600 (JASCO, Tokyo, Japan). A Verios 460L (FEI, Hillsboro, OR, USA) was used to take field emission scanning electron microscope (FE-SEM) images and also used to perform energy dispersive spectroscopy (EDS) mapping analysis.

### 2.5. Electrochemical Measurements

Electrochemical responses of the MnO_x_/rGO composites were measured using a three-electrode system. A 6 M KOH aqueous solution was used as the electrolyte, a platinum plate was used as the counter electrode, and Ag/AgCl was used as the reference electrode. The working electrode was prepared by mixing 80 wt% active material, 15 wt% activated carbon, and 5 wt% polyvinylidene fluoride (PVDF) in N-methyl-2-pyrrolidone (NMP). The slurry was then spread onto a glassy carbon electrode, which was used as the current collector. The electrode was then heated at 60 °C for 24 h to evaporate the solvent. The rate of total deposited mass throughout our study was 1.5 mg cm^−2^. To investigate the electrochemical performance of the electrodes, cyclic voltammetry (CV) and galvanostatic charge-discharge techniques were employed. Electrochemical performance in the three-electrode configuration was determined in a CH660D electrochemical station (CH Instruments, Inc., Austin, TX, USA).

## 3. Results and Discussion

### 3.1. Materials Characterization

**Figure 1:**
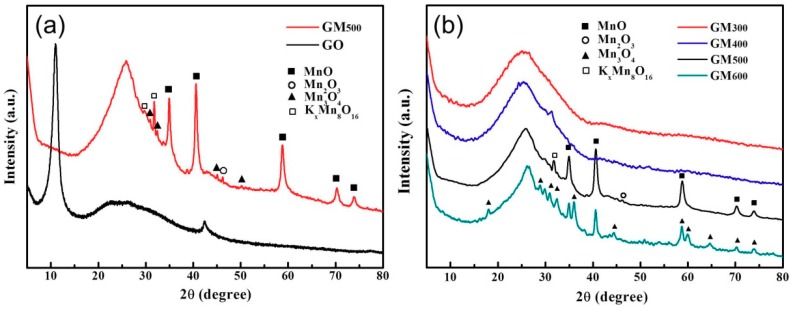
(**a**) X-ray diffraction (XRD) spectra of the graphene oxide (GO) and GM_500_; (**b)** Comparative XRD spectra of GM_300_, GM_400_, GM_500_, and GM_600_ (denoted the MnO_x_/rGO as GM_n_, where n is the annealing temperature).

The MnO_x_/rGO nanocomposites were synthesized using manganese nitrate and potassium permanganate as co-precursors for the MnO_x_. First, electrostatic binding of manganese (II) cations on the surface of GO was achieved. With the addition of potassium permanganate, permanganate (MnO_4_^−^) ions were bonded with Mn^2+^ ions. Subsequent annealing at 500 °C in an Ar atmosphere led to the resulting MnO_x_/K^+^-doped rGO composites. No washing was involved in this process; therefore, abundant K^+^ ions were introduced in the composite structure. The X-ray diffraction (XRD) patterns of the compositions and crystal structures of the MnO_x_/rGO nanocomposites and the GO are shown in Figure 1a. Sharp characteristic peaks corresponding to the MnO_x_ on the surface of the graphene appeared at 23.1°, 30.0°, 31.7°, 34.9°, 40.5°, 47.3°, 58.7°, 70.1°, and 73.7°, indicating the presence of mixed-valence MnO_x_ including MnO, Mn_2_O_3_, and Mn_3_O_4_, which was confirmed by Joint committee on powder diffraction standards (JCPDS) No. 07-0230 (MnO), JCPDS No. 00-041-1442 (Mn_2_O_3_), and JCPDS-International Center for diffraction data (ICDD) No. 00-07-0230 (Mn_3_O_4_), respectively. It was found that using the annealing temperature of 500 °C was beneficial for the development of the mixed-valence manganese oxides in this work (Figure 1b). At lower annealing temperatures, such as 300 °C or 400 °C, negligible peaks of manganese oxides were detected, indicating that such annealing temperatures were insufficient for the development of MnO_x_ crystals. In addition, when the annealing temperature reached 600 °C, most MnO_x_ were thermally converted to a Mn_3_O_4_ single phase [23]. The nominal peaks of K^+^-intercalated MnO_x_ structure (K_x_Mn_8_O_16_) was also detected alongside in case of the composite annealed at 500 °C.

**Figure 2:**
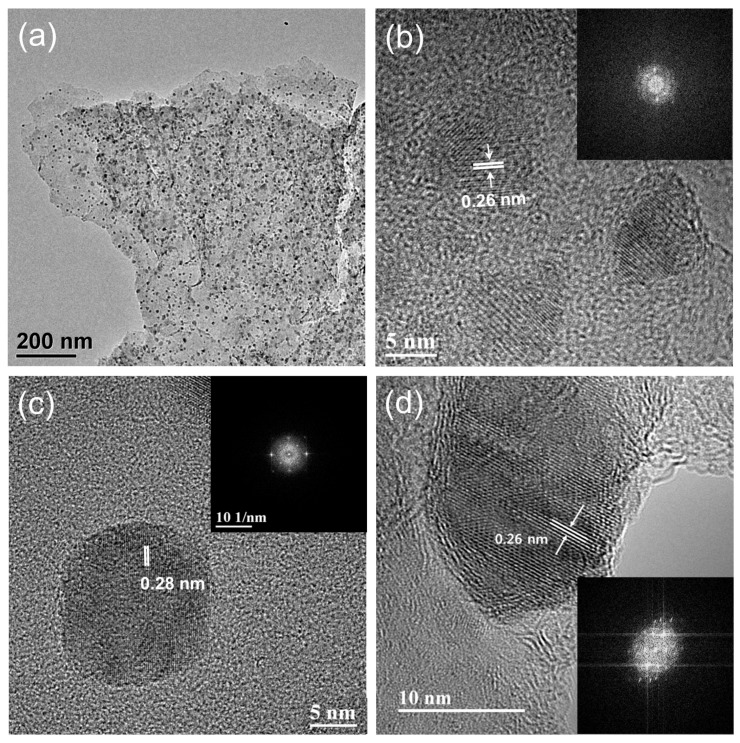
(**a**) A typical transmission electron microscopy (TEM) image of GM_500_, and the enlarged TEM images which reveal the structures of (**b**) MnO, (**c**) Mn_3_O_4_, and (**d**) Mn_2_O_3_.

Typical microstructures of the MnO_x_/rGO nanocomposites, investigated by TEM, are shown in Figure 2. As seen in Figure 2a, MnO_x_ nanoparticles in the range of 6 to 12 nm grew on the surface of the graphene sheets. Our proposed approach allowed us to achieve uniformly distributed MnO_x_ particles without nanoparticle aggregation. The loaded amount of the MnO_x_ on the surface of the graphene can be controlled by varying the amounts of precursors used for the growth of the MnO_x_. Figure 2b shows a lattice spacing of 2.6 nm, which corresponds to the (111) plane of MnO. Figure 2c shows the 0.28 nm lattice spacing of the (200) plane of Mn_3_O_4_. Figure 2d shows the 0.26 nm lattice spacing of the (222) plane of the Mn_2_O_3_. The MnO_x_ phase is found to be one of the following: MnO, Mn_2_O_3_, and Mn_3_O_4_. In addition, the nominal presence of the K^+^-intercalated MnO_x_ structures were observed through TEM analyses (Figure S1), which are in agreement with the XRD results. 

**Figure 3:**
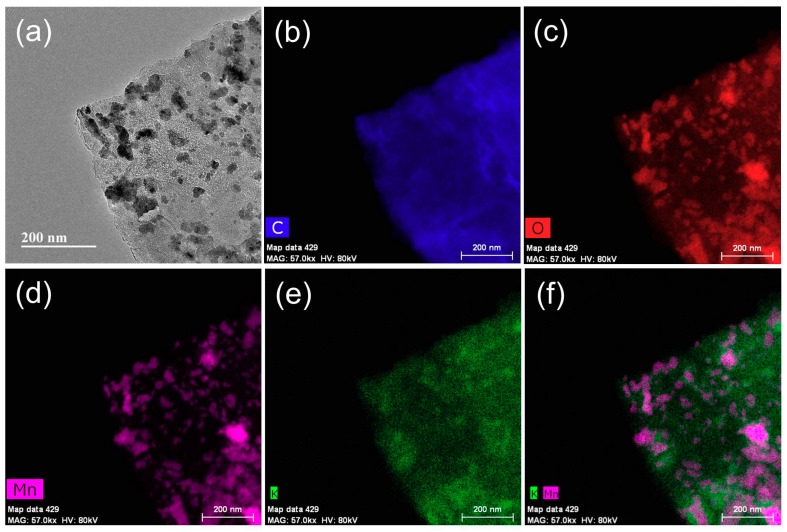
Energy dispersive spectroscopy (EDS) analysis of GM_500_ with TEM. (**a**) TEM image of GM_500_, elemental maps of (**b**) C, (**c**) O, (**d**) Mn, and (**e**) K, and the (**f**) Mn-K composite image.

Figure 3 displays the TEM-EDS maps showing the distributions of C, O, Mn, K, and K-Mn in combination. It also shows that the K^+^ ions were uniformly distributed on the rGO surface and near the grain boundary of the polymorphs of the MnO_x_. Also, a small amount of the K^+^ ions were detected within the MnO_x_ structure (Figure 3e,f). This is due to the small number of K^+^-intercalated MnO_x_ structures present in the composites, which is in accordance with the XRD study. During calcination at 500 °C, it is known that NO_3_^2−^ ions can easily evaporate, but K^+^ ions remain stably along the grain boundary of the polymorphs of MnO_x_, as well as on the surface of the rGO [24].

**Figure 4:**
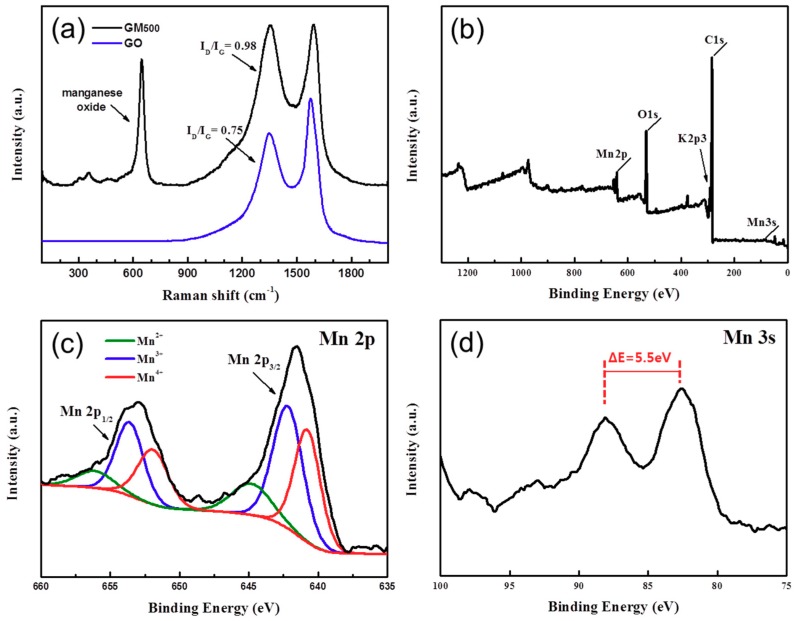
(**a**) Raman spectra of the GO and GM_500_. (**b**) X-ray photoelectron spectroscopy (XPS) survey scan of GM_500_. (**c**) Mn 2p region, and (**d**) Mn 3s region of GM_500_.

The Raman spectra of the GO and the MnO_x_/rGO nanocomposite are shown in Figure 4a. A D band at 1346 cm^−1^ and a G band at 1570 cm^−1^ were detected in the spectrum of GO. The characteristic D and G bands are observed for both the MnO_x_/rGO nanocomposite and the GO products. The intensity ratios of the D to G bands for GO and the MnO_x_/rGO nanocomposite show an obvious change: 0.75 and 0.98, respectively. The increasing value of the D/G intensity ratio of the MnO_x_/rGO nanocomposite in comparison to that of the GO is due to the reduced size of the sp^2^ domains and the creation of new graphitic domains with smaller sizes after thermal reduction at 500 °C [25]. In addition, the peaks at 639.7 cm^−1^ for the MnO_x_/rGO are assigned to the Mn–O stretching vibration in the basal plane of MnO_6_ and/or the symmetric stretching vibration of the MnO_6_ group [26]. 

The surface electronic state and the chemical bonding state of MnO_x_/rGO were analyzed in detail by XPS. As shown in Figure 4b, the survey XPS spectrum for the MnO_x_/rGO nanocomposite revealed the presence of carbon, manganese, potassium, and oxygen. In particular, the omission of the rinsing step after the reaction of the KMnO_4_ in the preparation of the MnO_x_/rGO nanocomposite may account for the presence of the K^+^ inside the MnO_x_/rGO after annealing. The C 1s peak originates from the graphene nanosheets. The Mn 2p peak was further inspected by high-resolution XPS analysis, as shown in Figure 4c. The two peaks at 641.5 eV and 653.2 eV can be assigned to Mn 2p_3/2_ and 2p_1/2_, respectively, which are characteristic of mixed-valence MnO_x_ [27,28]. The oxidation state of the manganese was confirmed by the multiplet splitting of the Mn 3s state. As shown in Figure 4d, the splitting width was 5.5 eV, which is in accordance with a previous report on the XPS spectrum of Mn_3_O_4_ [19]. Fourier transform infrared spectroscopy (FT-IR) was used to characterize the GO and the MnO_x_/rGO nanocomposites, and the results are shown in Figure S2. The GO showed a broad band at 3300–3700 cm^−1^ and a distinct band at 1620~1730 cm^−1^, corresponding to O–H and C=O, C–O respectively. After the composite was made, all bands decreased significantly, and a new peak, for the MnO_x_ band, at 600 cm^−1^ was confirmed [29,30,31]. 

### 3.2. Electrochemical Properties

**Figure 5:**
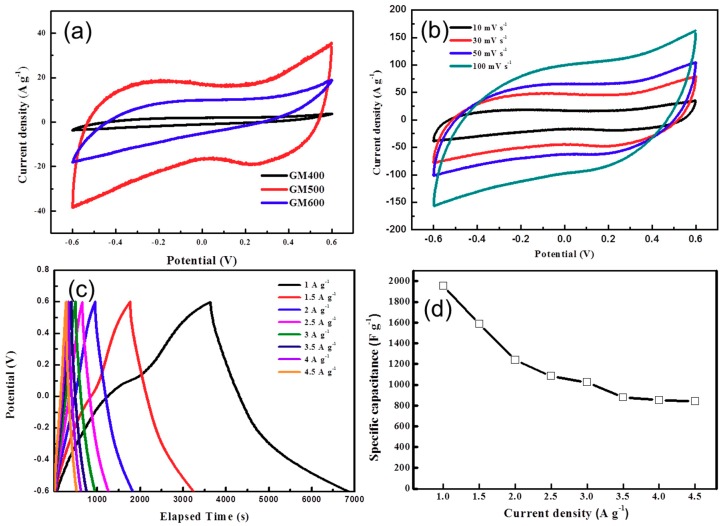
Cyclic voltammetry (CV) curves for (**a**) GM_400_, GM_500_, and GM_600_ at a scan rate of 10 mVs^−1^, (**b**) CV curves of GM_500_ at various scan rates, (**c**) Galvanostatic charge-discharge curves of GM_500_ at different current densities, and (**d**) Specific capacitance vs. current density plot for GM_500_.

To evaluate the temperature-dependent electrochemical behavior and quantify the capacitance performance of the MnO_x_/rGO nanocomposites, samples annealed at 400 °C, 500 °C, and 600 °C were prepared, and cyclic voltammetry (CV) measurements were taken at different scan rates under the operating potential of −0.6–0.7 V in a three-electrode system. The results are shown in Figure 5a. It can be observed that GM_500_ shows a larger capacitive area than either GM_400_ or GM_600_. This is because the pseudocapacitance is dependent on the structure of the metal oxide. Recently, it was established that a multivalent oxide system is capable of adsorbing anions as well as transporting the oxygens into vacant sites of manganese ions, thus promoting high redox reactions and faster electron transitions [32,33]. From the above XRD study, we observed that the crystal structure and the valence state of the resulting MnO_x_ and the following CV performances are dependent on the annealing temperature. GM_400_ showed very low capacitive current compared to GM_500_, probably due to the insufficient growth of the mixed-valence MnO_x_. GM_600_ also demonstrated an inferior performance to that of GM_500_. This is attributed to the reduced redox active formation of the Mn_3_O_4_, which could, in turn, result in a reduction of the capacitive current for the increased annealing temperature of 600 °C. However, upon annealing at 500 °C, various mixed-valence MnO_x_ phases, such as MnO, Mn_2_O_3,_ and Mn_3_O_4_, were generated, which is found to be beneficial for a larger capacitive current area.

A CV analysis of GM_500_ at different scan rates was carried out to investigate the current response of GM_500_. All of the CV curves had a quasi-rectangular shape for both low and high scan rates (Figure 5b). Typically, at the high scan rates—above 50 mV s^−1^—the CV curves are distorted from their rectangular shape. This indicates unbalanced ion diffusion with respect to the charging and discharging currents, caused by the polarization between the metal oxide and electrolyte with limited incubation of electrolyte inside the electrode materials. However, the graphs are almost the same with respect to the zero-current axis. To quantify the capacitance value of GM_500_, we conducted galvanostatic charge/discharge measurements. Figure 5c shows a faint hump in the 0.09 V signal during charging, and a slight bend appears in the discharging graph. This is due to the significance of the redox reaction during electrochemical performance, which varies due to the presence of the mixed-valence MnO_x_ and the abundant K^+^ ions. Figure 5d presents specific capacitance values calculated from the following equation:

(1) $$C_{s}=\frac{2i_{m}\int V\;dt}{V^{2}|\begin{array}{c} Vf\\ Vi \end{array}},$$

where C_s_ (F g^−1^) is the specific capacitance, *i_m_* = *I/m* (A g^−1^) is the current density, where *I* is the current and *m* is the mass of the active material, ∫Vdt is the integral current area, where *V* is the potential with initial and final values of *V_i_* and *V_f_*, respectively [34].

**Figure 6:**
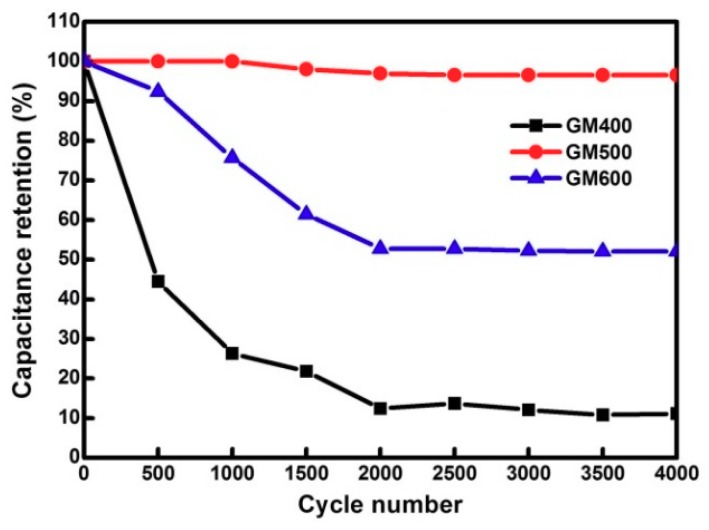
Cyclic stability of GM_400_, GM_500_, and GM_600_ at a current density of 1.0 A g^−1^.

The highest and lowest specific capacitance values were 1955.6 F g^−1^ and 840.7 F g^−1^ at current densities of 1 A g^−1^ and 4.5 A g^−1^, respectively. These high capacitive performances might be due to the presence of the K^+^ ions on the grain boundary of the MnO_x_ and the surface of the rGO, which were easily transported from the electrode to the electrolyte. In previous studies, the charge storage mechanisms of MnO_x_-based composites were controlled mainly by the intercalation/de-intercalation of alkali cations and the structural changes that occurred during the electrochemical performances [35]. In our study, most of the K^+^ ions were doped on the surface of the rGO, rather than intercalated within the MnO_x_. Since the K^+^ ions doped on the rGO induce an n-doping effect of graphene, the electrical conductivity of the rGO is expected to be enhanced [18]. In this regard, transportation of electrons from the redox center to the current collector can be promoted, resulting in an increase of capacitance performance. Moreover, the formation of mixed-valence MnO_x_ can result in superior performance of capacitive behavior, which benefits from the coexistence of aliovalent manganese cations that drive the defect-accelerated kinetics of the surface reactions. Both GM_400_ and GM_600_ show capacitive properties inferior to that of GM_500_, which indicates that during the annealing process, there is insufficient development of the mixed-valence MnO_x_ (Figures S3 and S4). In addition, GM_500_ exhibited the largest surface area, 210 m^2^ g^−1^, from Brunauer-Emmet-Teller (BET) analysis (Belsorp-max, Bel Japan Inc., Toyonaka, Japan) compared to that of GM_400_ and GM_600_, supporting the superior capacitive property of GM_500_ (Figure S5). The cyclic stability test results shown in Figure 6 reveal that the specific capacitance is retained as 96%, even after 4000 charge/discharge cycles. Based on these results, we conclude that GM_500_ is an effective material for use as a supercapacitor electrode. 

**Figure 7:**
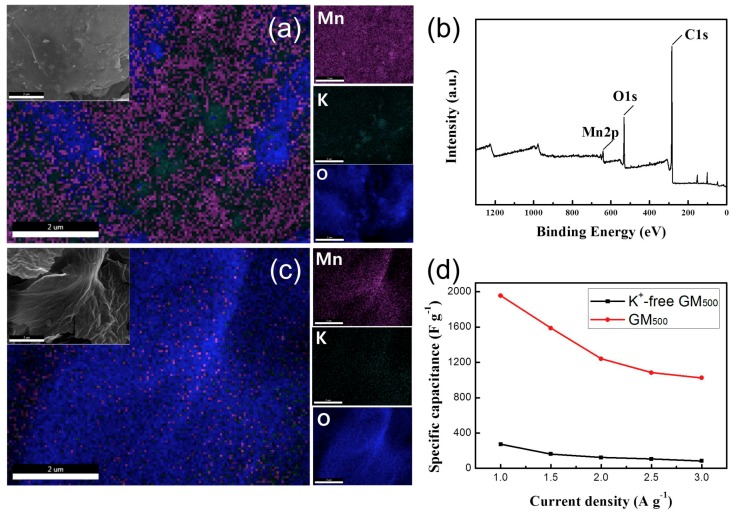
(**a**) Scanning electron microscopy (SEM) and EDS of GM_500_, (**b**) XPS survey scan of K^+^-free GM_500_, (**c**) SEM and EDS of K^+^-free GM_500_, (**d**) Specific capacitance versus current density of K^+^-free GM_500_.

To clarify the effect of the K^+^ ions, GM_500_ without K^+^ ions was also prepared. K^+^-free GM_500_ was prepared by repetitive washing after the reaction of the manganese precursors and subsequent annealing at 500 °C. From TEM and XRD studies, it is found that the resulting K^+^-free GM_500_ is composed of Mn_3_O_4_, without showing any multivalency (Figure S6). We employed scanning electron microscopy (SEM) and EDS mapping to compare the composition of elements before and after washing the GM_500_ (Figure 7). The EDS elemental mapping image of GM_500_ showed the K-edge signals of Mn, O, and K with atomic percentages of 22%, 39%, and 39%, respectively (Figure 7a). In contrast, the EDS mapping of K^+^-free GM_500_ indicates an increased amount of O and a dramatically decreased amount of K after washing. The atomic percentages of Mn, O, and K were 9%, 84%, and 7%, respectively (Figure 7c). Figure 7b shows the XPS survey scan of the K^+^-free GM_500_, presenting an absence of bonding associated with K ions. The above results indicate that the K^+^ ions are removed by washing. The electrochemical performances also show that K^+^-free GM_500_ is very inferior to GM_500_ with K^+^ doping, exhibiting a specific capacitance of 272 F/g at a current density of 1 A/g (Figure 7d). From these observations, we can conclude that the presence of K^+^ ions is essential for developing multi-valence MnO_x_ on the surface of the rGO upon annealing at a temperature of 500 °C, leading to the superior capacitive performance of the composite electrode.

## 4. Conclusions

In conclusion, we presented a facile synthesis of mixed-valence MnO_x_/K^+^-doped rGO nanocomposites for efficient supercapacitor electrodes. By using the electrostatic binding of the manganese precursor and KMnO_4_, and subsequent annealing, we successfully fabricated the MnO_x_/rGO composites with uniformly distributed MnO_x_ on the surface of K^+^-doped rGO. The abundant K^+^ ions remaining on the surface of the rGO helps to develop mixed-valence MnO_x_ and leads to a superior capacitive property by promoting a faster reversible reaction. Furthermore, it was found that an annealing temperature of 500 °C was suitable for sufficient growth of mixed-valence MnO_x_/rGO nanocomposites. The resulting composite materials yield a specific capacitance of 1955.6 F g^−1^ at 1 A g^−1^, which demonstrates that these K^+^-doped MnO_x_/rGO nanocomposites would be attractive for various energy applications.

## Supplementary Materials

The following are available online at https://www.mdpi.com/1996-1944/12/8/1245/s1, Figure S1: TEM image showing nominal presence of K^+^-intercalated MnO_x_ on the surface of the rGOs. The lattice spacing corresponds to (200) plane of K_x_Mn_8_O_16_. Figure S2: FT-IR spectra of the GO and the MnO_x_/rGO composites. Figure S3: (a) GCD curves of GM_400_; (b) specific capacitance variation of GM_400_ according to the change of current density; (c) GCD curves of GM_600_; (d) specific capacitance variation of GM_600_ according to the change of current density. Figure S4. Electrochemical impedance spectroscopy (EIS) plots of GM_400_, GM_500,_ and GM_600_. Figure S5. BET surface area analysis of the GM_500_. Figure S6. K^+^-free GM_500_ prepared from repetitive washing. (a) SEM; (b) TEM; (c) XRD data shows that the washed GM_500_ is composed of Mn_3_O_4_ single phase.
