# Supplementary material for: Effect of Potassium Ions on the Formation of Mixed-Valence Manganese Oxide/Graphene Nanocomposites
Source: Materials (Basel). 2019 Apr 16;12(8):1245. doi: 10.3390/ma12081245 (PMC6515087; doi:10.3390/ma12081245)
Supplement: Supplementary file 1 [file materials-12-01245-s001.pdf]

materials

Supplementary Materials

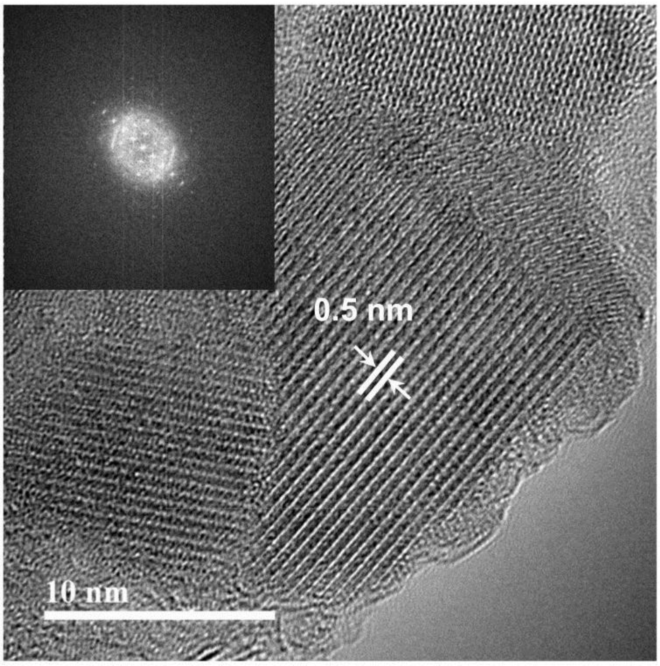

Figure S1. TEM image showing nominal presence of K+-intercalated MnO_x on the surface of the rGOs. The lattice spacing corresponds to (200) plane of $K_xMn_8O_{16}$.

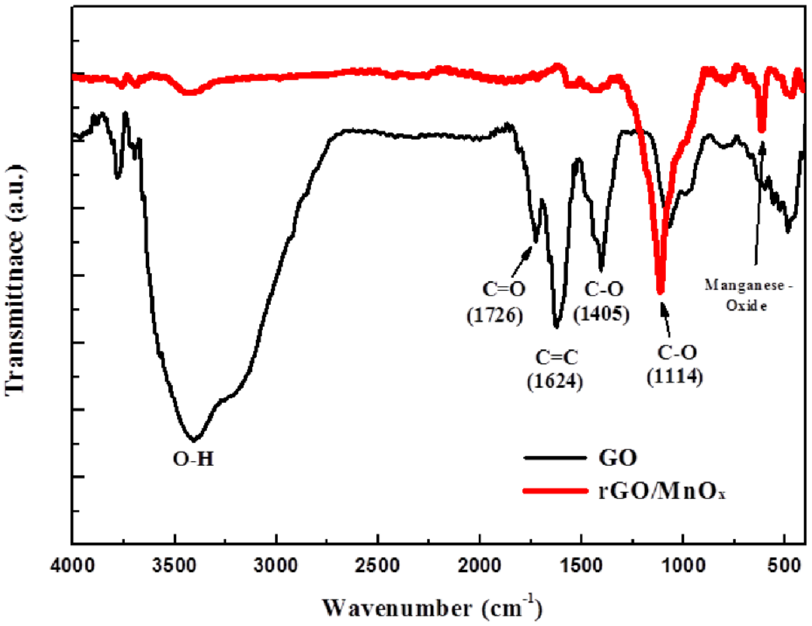

Figure S2. FT-IR spectra of the GO and the MnO_x/rGO composites.

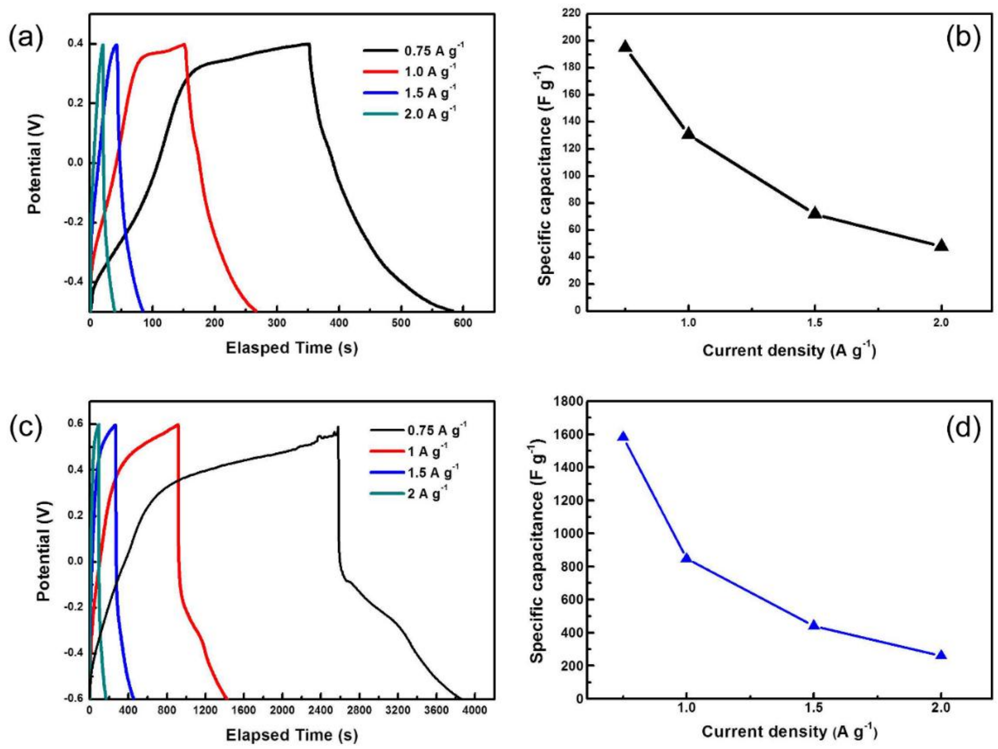

Figure S3. (**a**) GCD curves of GM_{400}; (**b**) specific capacitance variation of GM_{400} according to the change of current density; (**c**) GCD curves of GM_{600}; (**d**) specific capacitance variation of GM_{600} according to the change of current density.

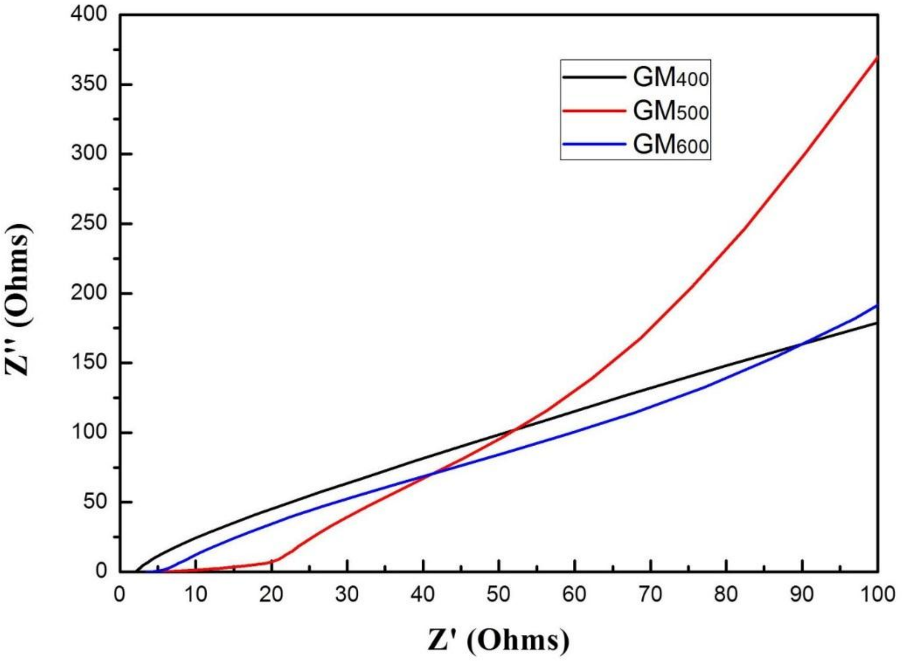

Figure S4. Electrochemical impedance spectroscopy (EIS) plots of GM_{400}, GM_{500}, and GM_{600}.

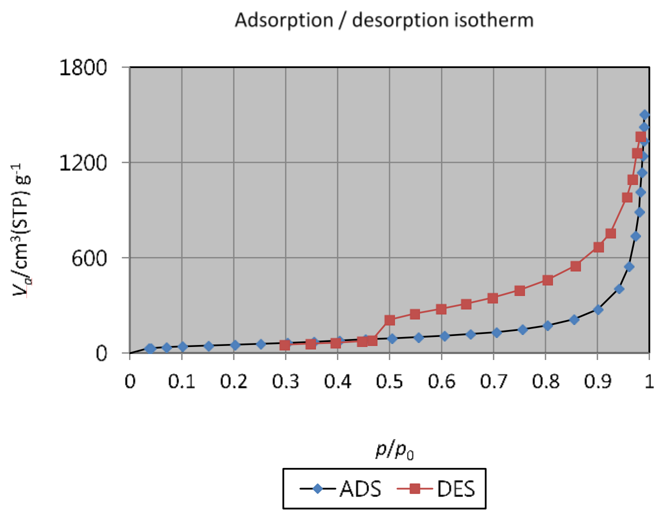

Vm			48.429 [cm^3(STP) g^{-1}]
$a_{s,BET}$			**210.79 [m^2 g^{-1}]**
C			29.529
Total pore volume(p/p_0=0.990)			2.2973 [cm^3 g^{-1}]
Average pore diameter			43.595 [nm]

Figure S5. BET surface area analysis of the GM_{500}.

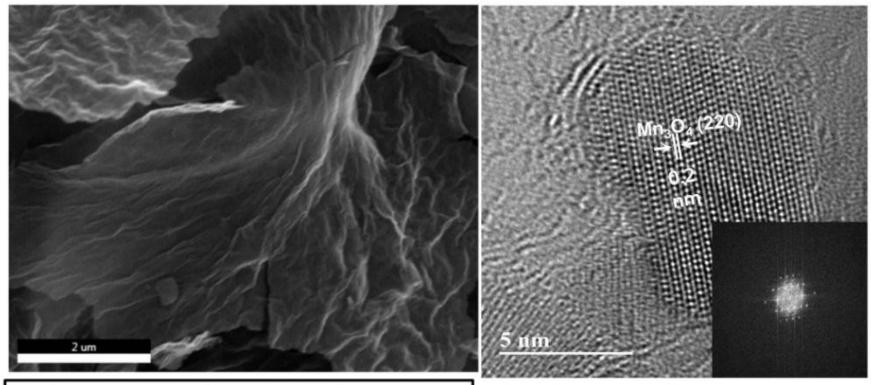

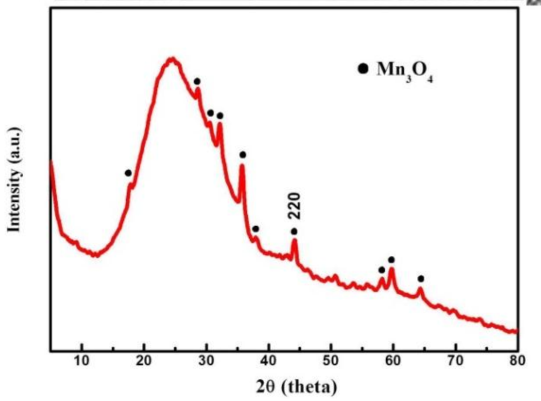

Table. The calculation of d-spacing from XRD pattern of w-GM500

	2θ from XRD pattern (degree)	d-spacing from XRD (Å)	d (nm)	from XRD card
wahing-GM 500	32.17	2.78	0.28	(103) Mn3O4
	35.66	2.52	0.25	(211) Mn3O4
	44.24	2.05	0.20	(220) Mn3O4
	59.79	1.55	0.16	(224) Mn3O4
	64.42	1.45	0.15	(314)Mn3O4

Figure S6. K^+-free GM_{500} prepared from repetitive washing. (**a**) SEM; (**b**) TEM; (**c**) XRD data shows that the washed GM_{500} is composed of Mn_3O_4 single phase.

© 2019 by the authors. Licensee MDPI, Basel, Switzerland. This article is an open access article distributed under the terms and conditions of the Creative Commons Attribution (CC BY) license (http://creativecommons.org/licenses/by/4.0/).
